# The Guiding Symptoms of Our Materia Medica

**Published:** 1890-07

**Authors:** 


THE GUIDING SYMPTOMS OF OUR MATERIA MEDICA. By C. Hering, M. D. Volume VIII. Philadelphia. Published by the Estate of Constantine Hering, 112 North Twelfth Street.

This noble volume, the eighth section of Dr. Hering's incomparable materia medica, is now before the profession. It contains 658 pages, and includes all the remedies in alphabetical order from Natrum-phosphoricum to Pulsatilla.

Its well-known arrangement of symptoms, each symptom standing alone, is in itself a recommendation, as it makes the finding of any particular indication a much easier task. Another admirable arrangement is the chapter upon sensations to be found under each remedy. This feature is an additional help in the search for the simillimum. Among the recent remedies introduced we find Cod-Liver Oil and Polygonum, or Smart Weed. Nothing, however, has been admitted that has not been proved. We consider it a reliable work, and make constant use of it in our own practice.

The delay in the appearance of the present volume is explained by the publisher to be due to the edition of the first four volumes having been exhausted, owing to the increased demand for the work, and the need to print them over again. The pages not having been stereotyped, it was necessary to set up every line anew.

